# Comparative genomic profiling of virulence genes in human clinical isolates of *Salmonella enterica serovars Typhimurium* and *Enteritidis*

**DOI:** 10.3389/fcimb.2025.1724145

**Published:** 2025-12-12

**Authors:** Hamid Reza Sodagari, Ihab Habib

**Affiliations:** 1 Independent Researcher, Perth, WA, Australia; 2 Veterinary Public Health Research Laboratory, Department of Veterinary Medicine, College of Agriculture and Veterinary Medicine, United Arab Emirates University, Al Ain, United Arab Emirates

**Keywords:** *Salmonella *typhimurium, *Salmonella *enteritidis, virulence genes, clinical isolates, comparative genomic

## Abstract

**Introduction:**

Non-typhoidal *Salmonella* are a significant global public health concern, which are responsible for considerable morbidity, mortality, and economic burden around the world. Among more than 2,600 *S. enterica* serovars Enteritidis and Typhimurium are the most prevalent in human infections globally. Identifying the genetic determinants that contribute to the pathogenic potential of these serovars is essential for developing prevention, control, and treatment strategies. The virulence mechanism for individual *Salmonella* serovars is still not well recognized. Diversity of virulence factors among serovars plays a critical role in disease severity and epidemiological success. Serovar-specific genes may influence infection strategies, host adaptation, and epidemiological patterns.

**Methods:**

Using public repositories of the NCBI Pathogen Detection database, this study aimed to determine genes that are shared and specific to each serovar and also identify significantly enriched genes in each serovar using statistical analysis.

**Results:**

While both serovars shared a conserved set of core virulence genes, significant serovar-specific differences were identified, which may reflect distinct strategies for host interaction, immune modulation, and nutrient acquisition.

**Conclusion:**

Our findings may have potential clinical and public health implications. Knowledge of serovar-specific virulence patterns may assist risk-based surveillance and targeted outbreak investigations. These findings enhance our understanding of *Salmonella* virulence profiling, particularly for two highly prevalent serovars in human infections, and may assist state, national, and international public health authorities in their efforts for future surveillance, risk assessment, and targeted intervention strategies.

## 1 Introduction

Non-typhoidal *Salmonella* are a significant global public health concern, which are responsible for considerable morbidity, mortality, and economic burden around the world (Balasubramanian et al., 2019; GBD 2017 Non-Typhoidal Salmonella Invasive Disease Collaborators, 2019). These pathogens are responsible for approximately 93.8 million gastroenteritis cases and 155,000 deaths annually worldwide (Majowicz et al., 2010). Human *Salmonella* infections are mostly associated with the consumption of contaminated food (Ehuwa et al., 2021); however, the infections are also acquired through direct and indirect contact with animals (Hoelzer et al., 2011). Although *Salmonella* infections are mostly self-limiting, invasive systemic diseases can occur in high-risk individuals (Smith et al., 2016; Zizza et al., 2024). Among more than 2,600 *S. enterica* serovars (Frasson et al., 2016), *S.* Enteritidis and *S.* Typhimurium are the most prevalent in human infections globally (Hendriksen et al., 2011), accounting for almost 60% of human salmonellosis worldwide (Hendriksen et al., 2011). In Australia, *S.* Typhimurium is the most prevalent serovar linked to human infections (Laidlow et al., 2022), while *S.* Enteritidis is among the top five reported *Salmonella* serovars (OzFoodNet Working Group, 2010). Most *S.* Enteritidis cases are travel-related, and only about 10% of the infections are locally acquired (OzFoodNet Working Group, 2015).

Identifying the genetic determinants that contribute to the pathogenic potential of these serovars is essential for developing prevention, control, and treatment strategies. The pathogenicity of *Salmonella enterica* is mediated by a diverse set of virulence factors, including the capsule, flagella, plasmids (Jajere, 2019), and Type III secretion systems (T3SS-1 and T3SS-2). The latter are encoded on *Salmonella* pathogenicity islands (SPI-1 and SPI-2) and function by delivering effector proteins into host cells, which facilitates invasion and intracellular survival (Zhao et al., 2025). Additional virulence determinants include adhesins, which mediate attachment to host epithelial surfaces (Ling et al., 2023) and iron acquisition systems, such as siderophore-mediated pathways, that enable survival in iron-limited host environments (Mey et al., 2021). The presence and functional diversity of these factors shape the virulence repertoire of individual isolates, affecting disease severity and epidemiological success (Cheng et al., 2019).

The virulence mechanism for individual *Salmonella* serovars is still not well recognized (Fenske et al., 2023). Previous study showed that serovars represent differences in pathogenesis and host preferences (Fenske et al., 2023). Diversity of virulence factors among serovars plays a critical role in disease severity and epidemiological success (Cheng et al., 2019). Previous comparative genomic investigations demonstrated that certain genes form a conserved “core virulome” across multiple serovars (Chaudhuri et al., 2013), while others are variably distributed or restricted to specific lineages (Jacobsen et al., 2011). These serovar-specific genes may influence infection strategies, host adaptation, and epidemiological patterns (Rakov et al., 2019). Although *S.* Typhimurium and *S.* Enteritidis share several virulence factors, they also have some differences in their pathogenic mechanisms. Both carry SPI-1 and SPI-2, which encode Type III secretion systems essential for host cell invasion and intracellular survival (Alghoribi et al., 2019). However, Typhimurium possesses broader SPI profiles, suggesting that this serovar may have higher pathogenic potential (Jin et al., 2024). On the other hand, Enteritidis has a more specialized repertoire, which is associated with iron acquisition and persistence. Added to that, this serovar harbors genes related to biofilm formation, enhancing its survival in the avian intestinal tract (Chen et al., 2021). Although *S.* Typhimurium, is derived from several food animal sources, *S.* Enteritidis is predominantly isolated from poultry, which shows this serovar has likely evolved adaptations that enhance its association with poultry hosts (Shah et al., 2017).

Although several previous studies focused on a lower number of isolates or single serovars, large-scale comparative studies of virulence gene content between *S.* Enteritidis and *S.* Typhimurium in specific geographic regions remain limited. Therefore, using public repositories of the National Center for Biotechnology Information (NCBI) Pathogen Detection database (https://www.ncbi.nlm.nih.gov/pathogens/), this study aimed to (i) identify prevalence of virulence-associated genes across *S.* Enteritidis and *S.* Typhimurium clinical human isolates from Australia, (ii) determine genes that are shared and specific to each serovar, and (iii) determine significantly enriched genes in each serovar using statistical analysis.

## 2 Materials and methods

### 2.1 Study design and data collection

The data analyzed in the present study were obtained from NCBI Pathogen Detection database (https://www.ncbi.nlm.nih.gov/pathogens/), which integrates genomic data submitted from various surveillance and research efforts worldwide. Data for clinical human *Salmonella enterica* isolates, comprising serovars Enteritidis and Typhimurium, were included in the present study. Clinical isolates refer to samples obtained from human patients during routine diagnostic testing or outbreak investigations. To reduce geographic heterogeneity and ensure epidemiological relevance to a specific region, we restricted our dataset to a single country and included only isolates from Australia collected between 2010 and 2024 for further analysis. After filtering and cleaning the dataset, including removal of duplicate entries and isolates with incomplete or inconsistent metadata, a total of 2,800 *S.* Enteritidis and 8,176 *S.* Typhimurium isolates were included in this study for further analysis.

### 2.2 Virulence gene extraction and processing

We extracted virulence genes for each isolate from the downloaded dataset. Only genes annotated as “COMPLETE” (full-length gene sequences without truncations) in the dataset were considered for further analysis, and genes annotated as “PARTIAL (incomplete or truncated sequences) were excluded. After that, a binary matrix from the extracted data was generated. Genes were coded as 1 (present) or 0 (absent) for each isolate. The matrix was applied for further descriptive and statistical analyses. All analysis was performed using R software (Version 4.5.0 (2025-04-11)) (R Core Team, 2025), within the RStudio platform (2024.09.0 Build 375 ^©^ 2009–2024 Posit Software, PBC).

### 2.3 Descriptive analysis of virulence genes

Prevalence of each virulence gene was calculated across all isolates in each serovar. Moreover, the number of common and serovar-specific genes (genes shared and unique to each serovar) was also determined using a Venn diagram by the ggVennDiagram R package.

### 2.4 Statistical analysis of virulence gene enrichment

To assess serovar-specific enrichment (Typhimurium versus Enteritidis), a 2×2 contingency table was constructed for each gene, comparing presence versus absence across the two serovars. Fisher’s exact test was applied to evaluate whether differences in gene prevalence were statistically significant. To explain multiple comparisons, p-values were adjusted using the False Discovery Rate (FDR) method. This controls the expected proportion of false positives among the genes identified as significantly enriched, reducing the chance of type I errors that can occur when testing many genes simultaneously. Genes with an FDR-adjusted p-value <0.05 were considered significantly enriched.

Added to that, we also calculated the magnitude of enrichment. Odds ratios (ORs) were calculated for each gene to estimate the likelihood of gene presence in one serovar compared to the other. For genes with zero counts in any category, a Haldane-Anscombe correction was applied, which adds 0.5 to all cells in a contingency table when a gene has zero counts. This avoids infinite OR estimates. We computed 95% Confidence Interval (CI) for each OR and also reported absolute differences in gene prevalence between serovars.

Significantly enriched genes were visualized in a barplot using ggplot2 R package. To provide the magnitude and direction of enrichment of the virulence genes, a forest plot showing the OR and 95% CI for each gene was also constructed. OR >1 indicates enrichment in Typhimurium, while OR <1 shows enrichment in Enteritidis.

### 2.5 Functional annotation of significantly enriched genes

To provide biological context for the significantly enriched genes, functional annotation was performed using the Virulence Factor Database (VFDB). For each gene, information on gene name, function, and mechanism of action was extracted and added to a table. For genes with no information found in the VFDB, the mechanism of action was obtained from published functional studies.

## 3 Results

### 3.1 Descriptive analysis

Across all 10,976 isolates (*S.* Enteritidis = 2,800 and *S.* Typhimurium = 8,176), a total of 26 virulence-associated genes were identified in the dataset. This number represents complete genes annotated as virulence factors in the NCBI Pathogen Detection database and does not reflect the total number of virulence genes present in individual genomes, as partial or unannotated genes were excluded.

### 3.2 Prevalence of virulence genes in *S. *enteritidis and *S. *typhimurium isolates

**Figure 1:**
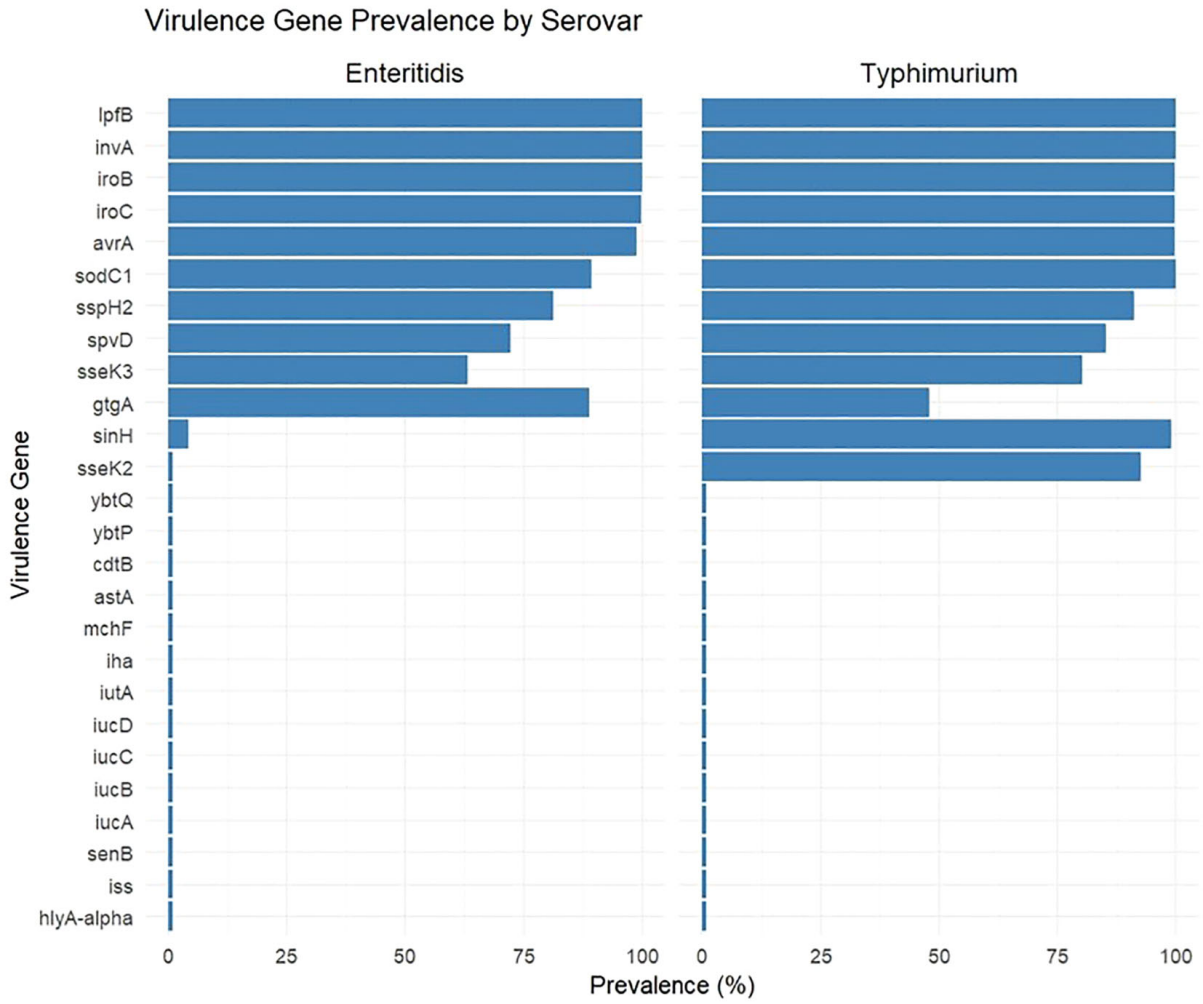
Prevalence of virulence genes among *S.* Enteritidis (n = 2,800) and *S.* Typhimurium (n = 8,176) clinical isolates.

Our results showed that the prevalence of virulence genes varied between the two serovars (**Figure 1**, **Supplementary Table S1**). Several genes, including *invA* (T3SS structural protein, invasion)*, avrA* (T3SS effector, immune modulation)*, iroB* and *iroC* (siderophore-mediated iron acquisition), and *lpfB* (fimbrial subunit, intestinal adhesion) were detected to be present in almost all *S.* Enteritidis and *S.* Typhimurium isolates.

In *S.* Enteritidis, genes such as *sodC1* (89.4%), *sspH2* (81.1%), *gtgA* (88.8%), *spvD* (72.1%), and *sseK3* (63.1%) were present in a considerable number of isolates, while others, such as *sinH* (4.3%), were identified in much lower rates. Several genes associated with iron acquisition or toxins, and genes such as *iucA–D, astA*, and others were completely absent or detected at very low prevalence (<1%) in *S.* Enteritidis.

On the other hand, *S.* Typhimurium exhibited a broader distribution of virulence factors. High prevalence rates were identified for genes such as *sodC1* (99.9%), *sinH* (98.9%), *sspH2* (91.0%), *sseK2* (92.4%), *sseK3* (80.3%), and *spvD* (85.1%). Moreover, *gtgA* was less frequent in *S.* Typhimurium (47.9%) compared with *S.* Enteritidis (88.8%). Our results also showed that some genes, such as *ybtP* and *ybtQ* although detected in very low frequencies (<1%) in *S.* Enteritidis isolates, were completely absent in *S.* Typhimurium isolates.

### 3.3 Shared and serovar-specific virulence genes

**Figure 2:**
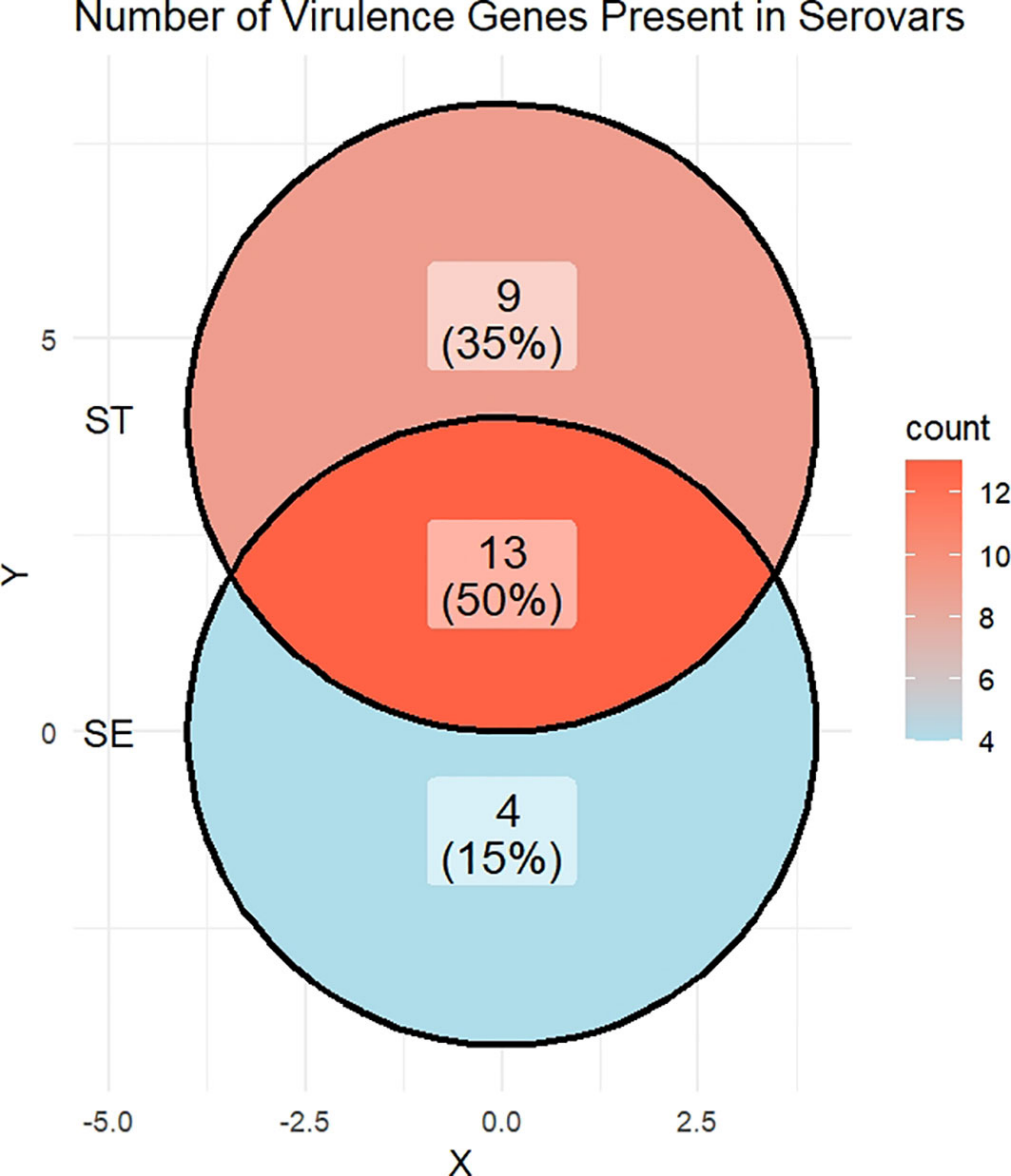
Venn diagram shows number (%) of shared and unique virulence genes in each serovar. ST: *S.* Typhimurium; SE: *S.* Enteritidis.

The distribution of common and serovar-specific virulence genes in *S.* Enteritidis and *S.* Typhimurium is shown in **Figure 2**. Of the 26 virulence genes analyzed, 13 genes (50%) were common to both *S.* Enteritidis and *S.* Typhimurium. This shared set included several *Salmonella* virulence factors, including *invA*, *lpfB*, *sinH* (structural/adhesion), *iroB*, *iroC* (siderophore-mediated iron acquisition), *sodC1* (oxidative stress defense), *avrA, sseK3, sspH2, gtgA* (T3SS effectors, immune modulation), *spvD* (Plasmid-encoded, immune evasion/intracellular survival), and *astA, mchF* (toxin/secondary metabolite related). Added to that, four genes (15%) were uniquely detected in *S.* Enteritidis. These included *ybtP, ybtQ* (yersiniabactin siderophore transport, iron acquisition)*, cdtB* (cytolethal distending toxin, host cell cycle arrest), and *iha* (adhesin, intestinal colonization), which were absent in all *S.* Typhimurium isolates. The other nine genes (35%) were specific to *S.* Typhimurium, including *sseK2* (T3SS effector, immune modulation), *iss* (complement resistance, serum survival)*, iucA, iucB, iucC, iucD, iutA* (aerobactin siderophore system, iron acquisition)*, hlyA-α* (α-hemolysin, pore-forming toxin), and *senB* (enterotoxin, diarrheagenic effect), none of which were detected in *S.* Enteritidis. **Table 1** shows both shared virulence genes (common to the two serovars) and unique virulence genes in each serovar.

**Table 1:** Shared and unique virulence genes in each serovar.

Shared genes	*avrA, invA, iroB, iroC, lpfB, sinH, sodC1*, sp*vD, sseK3, sspH2, gtgA, astA, mchF*,
*S.* Typhimurium-unique genes	*sseK2, iss, iucA, iucB, iucC, iucD, iutA, hlyA.alpha, senB*
*S.* Enteritidis-unique genes	*ybtP, ybtQ, cdtB, iha*

### 3.4 Serovar-specific enrichment of virulence genes

We analyzed the prevalence of 26 virulence genes across *S.* Enteritidis (n = 2,800) and *S.* Typhimurium (n = 8,176) isolates to identify genes significantly enriched in one serovar. A combination of Fisher’s exact test and FDR adjustment was applied, and enrichment magnitude was quantified using ORs with 95% CI and absolute prevalence differences (**Table 2**). The results showed 11 genes were significantly enriched in one serovar (adj. p < 0.05).

**Table 2:** Serovar-specific enrichment of virulence genes in *S.* Enteritidis and *S.* Typhimurium.

Gene	Prevalence (%) SE	Prevalence (%) ST	OR	OR – lower confidence limit	OR – Upper confidence limit	Absolute prevalence difference	p_value	Adjusted p_value	Enriched in
*avrA*	98.82	99.71	4.05	2.39	6.87	0.89	2.53E-07	6.58E-07	Typhimurium
*invA*	99.89	99.98	4.38	0.73	26.25	0.08	0.109	0.202	None
*iroB*	100	99.73	0.06	0	1.07	0.27	0.00238	0.00562	Enteritidis
*iroC*	99.68	99.73	1.2	0.55	2.6	0.05	0.68	1	None
*lpfB*	99.89	99.99	8.77	0.91	84.33	0.09	0.0537	0.116	None
*sinH*	4.29	98.87	1962.42	1490.45	2583.84	94.59	0	0	Typhimurium
*sodC1*	89.36	99.85	81.03	45.42	144.55	10.5	3.82E-163	2.49E-162	Typhimurium
*spvD*	72.11	85.1	2.21	1.99	2.45	13	8.69E-50	3.77E-49	Typhimurium
*sseK2*	0	92.43	68328.58	4267.43	1094053	92.43	0	0	Typhimurium
*sseK3*	63.14	80.28	2.38	2.16	2.61	17.14	1.32E-70	6.85E-70	Typhimurium
*sspH2*	81.14	91.02	2.36	2.09	2.66	9.88	1.64E-41	6.10E-41	Typhimurium
*gtgA*	88.75	47.86	0.12	0.1	0.13	40.89	0	0	Enteritidis
*iss*	0	0.01	1.03	0.04	25.23	0.01	1	1	None
*astA*	0.04	0.02	0.68	0.06	7.56	0.01	1	1	None
*iucA*	0	0.02	1.71	0.08	35.69	0.02	1	1	None
*iucB*	0	0.02	1.71	0.08	35.69	0.02	1	1	None
*iucC*	0	0.02	1.71	0.08	35.69	0.02	1	1	None
*iucD*	0	0.02	1.71	0.08	35.69	0.02	1	1	None
*iutA*	0	0.02	1.71	0.08	35.69	0.02	1	1	None
*hlyA.alpha*	0	0.01	1.03	0.04	25.23	0.01	1	1	None
*mchF*	0.04	0.01	0.34	0.02	5.48	0.02	0.445	0.723	None
*senB*	0	0.01	1.03	0.04	25.23	0.01	1	1	None
*ybtP*	0.82	0	0.01	0	0.12	0.82	2.11E-14	6.11E-14	Enteritidis
*ybtQ*	0.82	0	0.01	0	0.12	0.82	2.11E-14	6.11E-14	Enteritidis
*cdtB*	0.07	0	0.07	0	1.43	0.07	0.0651	0.13	None
*iha*	0.04	0	0.11	0	2.8	0.04	0.255	0.442	None

Genes significantly enriched in *S.* Typhimurium included *avrA, sseK2*, *sseK3, sspH2* (T3SS effectors, immune modulation), *spvD* (Plasmid-encoded, immune evasion/intracellular survival), *sinH* (structural/adhesion), and *sodC1* (oxidative stress defense). Some of these genes were present in almost all *S.* Typhimurium isolates but were rare or absent in *S.* Enteritidis. For example, *sinH* was detected in 98.9% of *S.* Typhimurium versus 4.3% of *S.* Enteritidis (OR = 1962.4; 95% CI: 1490.5–2583.8; absolute prevalence difference = 94.6%), and *sseK2* was present in 92.4% of *S.* Typhimurium but absent in *S.* Enteritidis (OR = 68,328.6; 95% CI: 4,267.4–1,094,053; absolute prevalence difference = 92.4%). Other significantly enriched genes, including *sodC1*, sp*vD, sseK3, sspH2*, and *avrA*, showed different ORs (2.21-4.05) with highly significant FDR-adjusted p-values (**Table 2**).

Genes significantly enriched in *S.* Enteritidis included *gtgA* (T3SS effector, immune modulation), *ybtP, ybtQ*, and *iroB* (siderophore-mediated iron acquisition). Our findings showed that, *gtgA* was detected in 88.8% of *S.* Enteritidis isolates compared with 47.9% of *S.* Typhimurium (OR = 0.12; 95% CI: 0.10–0.13; absolute prevalence difference = 40.9%), whereas *ybtP* and *ybtQ* were exclusively present in *S.* Enteritidis (prevalence = 0.82%) and absent in *S.* Typhimurium (OR = 0.01; 95% CI: 0–0.12; absolute prevalence difference = 0.82%). *iroB* was also significantly enriched in *S.* Enteritidis (OR = 0.06; 95% CI: 0–1.07; absolute prevalence difference = 0.27%) (**Table 2**).

The other genes, such as *invA, iroC, lpfB, astA, iss, senB, iucC*, *cdtB, iha*, and others, showed no significant differences between serovars after FDR adjustment (adj. p > 0.05) (**Table 2**).

**Figure 3:**
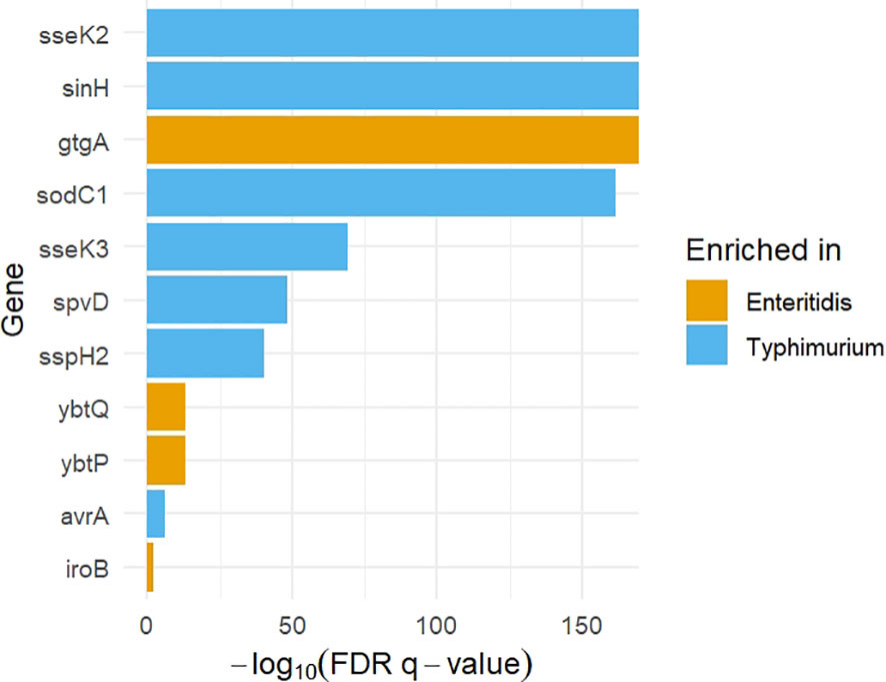
Significantly enriched virulence genes in *S.* Enteritidis and *S.* Typhimurium. Bar height represents statistical significance (–log_10_ FDR-adjusted p-value), and color indicates the serovar in which each gene is enriched.

**Figure 4:**
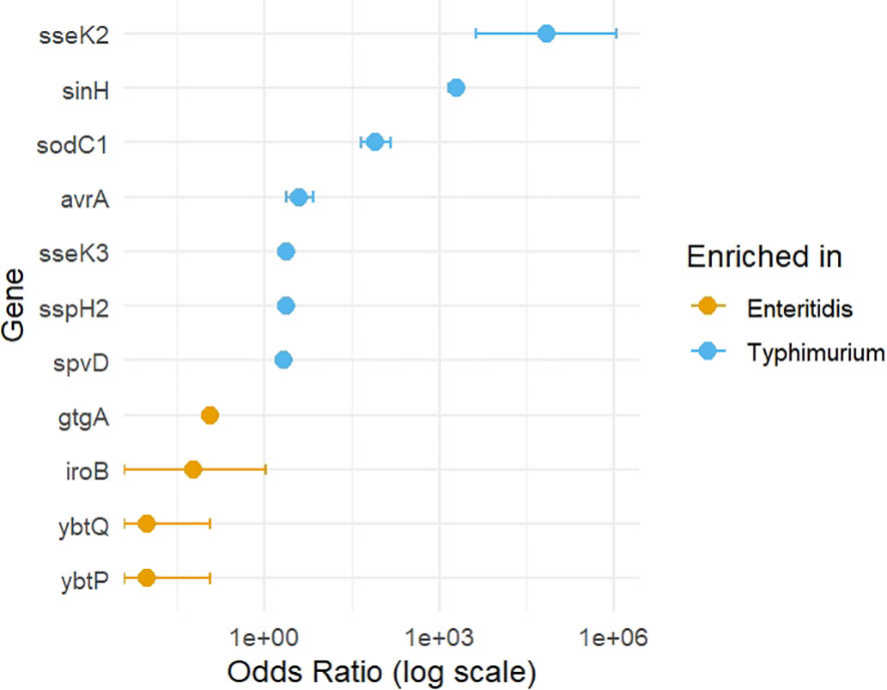
Forest plot shows ORs and 95% CIs for significantly enriched virulence genes in *S.* Enteritidis and *S.* Typhimurium. Points: ORs; Horizontal error bars: 95% CIs. ORs >1 indicate enrichment in Typhimurium, while ORs <1 indicate enrichment in Enteritidis.

The distribution of significantly enriched genes is shown in **Figure 3**. In addition, the forest plot represents ORs with 95% CIs, which provides a clear visualization of the magnitude and direction of serovar-specific enrichment (**Figure 4**).

### 3.5 Functional annotation of significantly enriched genes

To identify biological roles of the 11 genes identified as significantly enriched in one serovar, they were annotated using the Virulence Factor Database (VFDB) (**Table 3**).

**Table 3:** Functional annotation of significantly enriched genes.

Serovar	Gene	Function	Mechanism of action
Typhimurium	*avrA*	T3SS effector (acetyltransferase)	Invasion/host immune modulation
Typhimurium	*sinH*	Intimin-like protein	Adhesion
Typhimurium	*sodC1*	Superoxide dismutase (Cu-Zn)	Immune evasion (oxidative stress)
Typhimurium	*spvD*	Plasmid-encoded virulence protein	Immune evasion/intracellular survival
Typhimurium	*sseK2*	T3SS effector	Immune evasion/host signaling modulation
Typhimurium	*sseK3*	T3SS effector (Arginine N-Glycosyltransferase)	Immune evasion/host signaling modulation
Typhimurium	*sspH2*	T3SS effector (novel E3 ubiquitin ligase)	Immune evasion/host signaling modulation
Enteritidis	*gtgA*	SPI-2 T3SS effector	Immune evasion/host signaling modulation
Enteritidis	*ybtP*	Yersiniabactin transporter	Iron acquisition/siderophore system
Enteritidis	*ybtQ*	Yersiniabactin transporter	Iron acquisition/siderophore system
Enteritidis	*iroB*	Putative glycosyltransferase	Iron acquisition/siderophore system

Several genes enriched in *S.* Typhimurium, including *avrA, sseK2*, and *sseK3* encode Type III secretion system effectors, which are involved in host immune modulation. The gene *sspH2*, which is an E3 ubiquitin ligase, contributes to host cell signaling, while *spvD*, a plasmid-encoded virulence effector, promotes intracellular survival. *sinH*, an adhesion factor, facilitates host cell attachment. Moreover, *sodC1*, a superoxide dismutase, plays an important role in detoxifying reactive oxygen species, which enhances bacterial survival under oxidative stress.

In *S.* Enteritidis, enriched genes include *gtgA*, a Type III secretion effector mediating immune modulation. In addition, *ybtP* and *ybtQ* genes encode proteins involved in siderophore transport for iron acquisition. Added to that, *iroB*, also enriched in *S.* Enteritidis, participates in siderophore biosynthesis. This gene also supports iron uptake in host environments.

## 4 Discussion

In this study, we extracted and analyzed the virulence gene profiles using the NCBI Pathogen Detection database. We included 10,976 clinical *Salmonella enterica* isolates from Australia, focusing on the two most clinically prevalent serovars, *S.* Enteritidis (n = 2,800) and *S.* Typhimurium (n = 8,176). This study illustrates the shared virulence factors between these two serovars and provides a systematic comparison of serovar-specific virulence patterns that differentiate their pathogenic potential.

Our results revealed that a subset of virulence genes, including *invA*, *iroB*, *iroC*, *lpfB*, *sodC1*, *spvD*, *sseK3*, *sspH2*, and *gtgA*, were highly prevalent in both serovars, reflecting a conserved core of *Salmonella* virulence factors. These factors are critical for pathogenic processes such as invasion, adhesion, iron acquisition, intracellular survival, and immune modulation (Zhao et al., 2025). The presence of these core genes in the majority of isolates underscores their essential role in *Salmonella* pathogenicity.

Our results also showed that some of the virulence genes have a much higher prevalence in only one of the serovars. *S.* Typhimurium was more enriched for *sinH* and *sseK2*. The *sinH* gene encodes an autotransporter protein. This promotes the adhesion of *Salmonella* to host cells, which facilitates their invasion (Li et al., 2021). The *sseK2* is an important SPI-2 virulence gene for *Salmonella* pathogenicity that contributes to immune evasion by modulating host signaling pathways. Although the mechanism of this gene in bacterial virulence is not yet fully understood (Zhang et al., 2019), a previous study showed that the absence of *sseK2* reduces *S.* Typhimurium virulence, affecting its pathogenicity both *in vitro* and *in vivo* (Zhang et al., 2019). Considering the high prevalence of this gene in *S.* Typhimurium isolates, at least in this study, further research is required to identify the fundamental role of this important gene in the pathogenicity of *S.* Typhimurium.

On the other hand, *S.* Enteritidis showed a higher prevalence rate in *gtgA* compared to *S.* Typhimurium, reflecting that it may be considered as a core virulence gene playing a critical role in *S.* Enteritidis pathogenicity. This gene encodes a zinc metalloprotease effector protein that *Salmonella* secretes during infection to suppress the host immune response (Jennings et al., 2018).

Moreover, the *ybtP* and *ybtQ* genes, although of low prevalence, were found only in *S.* Enteritidis isolates in the present study. These yersiniabactin virulence genes are linked to immune modulation and iron acquisition, which facilitate iron acquisition, promoting *Salmonella* survival in low iron conditions (McMillan et al., 2020). This finding indicates that this serovar may rely more on nutrient acquisition systems and selective effector functions for successful infection. However, more studies are required to shed more light on this hypothesis.

Our findings also provide a comprehensive view of gene sharing between serovars. Approximately 50% of virulence genes were common, 35% were specific to *S.* Typhimurium, and 15% were specific to *S.* Enteritidis. These patterns suggest that while the two serovars share a conserved pathogenic core, evolutionary divergence has shaped serovar-specific virulence repertoires that may influence infection outcomes and clinical presentations (Wang et al., 2023).

Our analysis revealed distinct patterns of virulence gene enrichment between *S.* Typhimurium and *S.* Enteritidis. The results revealed that, out of 26 virulence genes, 11 showed significant serovar-specific enrichment, which indicates that certain virulence genes are associated with one serovar over the other.

*S.* Typhimurium showed enrichment of several genes, including *sinH, sseK2, sseK3, sspH2, avrA*, sp*vD*, and *sodC1*. These genes are involved in adhesion, invasion, host immune modulation, and intracellular survival activity. Our results also indicated that *sinH* and *sseK2* were present in the vast majority of *S.* Typhimurium isolates but were almost absent in *S.* Enteritidis. This indicates that there might be a strong serovar-specific signature. The presence of *sinH*, which encodes an autotransporter involved in adhesion and immune evasion (Li et al., 2021), and *sseK2*, a Type III secretion system effector implicated in host cell apoptosis and NF-κB inhibition (Araujo-Garrido et al., 2020), suggests that *S.* Typhimurium may possess enhanced mechanisms for intracellular persistence and immune modulation relative to *S.* Enteritidis. Other genes, such as *sspH2, avrA*, sp*vD*, and *sodC1* were moderately enriched in *S.* Typhimurium. This suggests that *S.* Typhimurium possesses a unique set of genes that may support its wider host range (Uzzau et al., 2000) and virulence potential.

Based on our results, *S.* Enteritidis showed significant enrichment of *gtgA, ybtP, ybtQ*, and *iroB*. According to our results, *ybtP* and *ybtQ* genes were identified only in *S.* Enteritidis. This suggests that this serovar may use different iron-acquisition strategies, which could influence its survival and replication in different host environments. Enrichment of *gtgA*, an effector that modulates host inflammatory responses (Sun et al., 2016), may further contribute to *S.* Enteritidis’s pathogenicity and adaptation to specific hosts.

Our analysis showed that serovar-specific enrichment may align with some separate biological virulence mechanisms in the two serovars. The pathogenicity of *S.* Typhimurium may be more associated with modulation of host immune responses and intracellular persistence, while *S.* Enteritidis pathogenicity may be more related to prioritizing iron acquisition and selective immune modulation. Further studies are required to reinforce our findings.

This study has several limitations. The study relied on publicly available genomic data from the NCBI database, which may introduce sampling bias due to uneven representation of isolates across geographic regions and time periods. In addition, our analysis was limited to genes annotated as “COMPLETE,” which means potential missing of new virulence determinants. Moreover, the present study only focused on the presence/absence of genes and did not consider expression levels, regulatory interactions, or environmental factors that influence gene activity. Finally, clinical metadata, such as patient outcomes or infection sites, were not available, limiting our ability to directly correlate virulence gene profiles with clinical severity.

## 5 Conclusion

In conclusion, this study provides a comparative analysis of virulence gene profiles in *S.* Enteritidis and *S.* Typhimurium clinical isolates from Australia. While both serovars shared a conserved set of core virulence genes, significant serovar-specific differences were identified, which may reflect distinct strategies for host interaction, immune modulation, and nutrient acquisition. Our findings may have potential clinical and public health implications. Knowledge of serovar-specific virulence patterns may assist risk-based surveillance and targeted outbreak investigations. These findings enhance our understanding of *Salmonella* pathogenicity, particularly for two highly prevalent serovars in human infections, and may assist state, national, and international public health authorities in their efforts for future surveillance, risk assessment, and targeted intervention strategies.

## Data Availability

The original contributions presented in the study are included in the article/**Supplementary Material**. Further inquiries can be directed to the corresponding authors.
